# Geographic Ecological Momentary Assessment (GEMA) of environmental noise annoyance: the influence of activity context and the daily acoustic environment

**DOI:** 10.1186/s12942-020-00246-w

**Published:** 2020-11-23

**Authors:** Xue Zhang, Suhong Zhou, Mei-Po Kwan, Lingling Su, Junwen Lu

**Affiliations:** 1 School of Geography and Planning, Sun Yat-Sen University, Guangzhou, China; 2 Guangdong Provincial Engineering Research Center for Public Security and Disaster, Guangzhou, China; 3 Department of Geography and Resource Management, The Chinese University of Hong Kong, Shatin, Hong Kong China; 4 Institute of Space and Earth Information Science, The Chinese University of Hong Kong, Shatin, Hong Kong China; 5 Department of Human Geography and Spatial Planning, Utrecht University, 3584 CB Utrecht, Netherlands

**Keywords:** Environmental noise, Real-time annoyance, Perception, GEMA, Activity place

## Abstract

**Background:**

Noise annoyance is considered to be the most widespread and recognized health effect of environmental noise. Previous research is mostly based on the static study of residential environmental noise, but few studies have focused on the effects of noise exposure in different activity contexts on real-time annoyance. The two deficiency are that they neglect the influence of activity context besides residence and fail to reflect the difference of time-scale effect of noise influence.

**Methods:**

Using portable noise and air sensors, GPS-equipped mobile phones, questionnaire survey, and geographic ecological momentary assessment (GEMA), this paper measured the environmental noise and real-time noise annoyance of participants at different activity places. Hierarchical logistic regression models were used to examine the effects of environmental noise on people’s real-time annoyance. The paper further considered the influence of the geographic context of the activity places and daily acoustic environment on participants’ real-time annoyance. Further, a nonlinear regression model was constructed using Random Forest to further examine the nonlinear relationship between environmental noise and real-time annoyance.

**Results:**

The results showed that: (1) the average cumulative equivalent sound level during was 55 dB (A) when the participants responded to the EMA surveys; (2) Only the temperature of activity places had an influence on momentary annoyance and the higher the temperature, the more likely participants were annoyed; (3) Participants with higher perception of noise pollution in residential communities were more likely to be annoyed. However, participants with higher daily exposure to noise were less likely to feel annoyed; (4) The threshold value of the effect of noise on real-time annoyance was 58 dB (A) to 78 dB (A).

**Conclusions:**

These findings can guide the development of urban planning and environmental noise standards and also provide a reference for noise barrier requirements for different activity places.

## Background

The effect of the environment on human health has been a major concern in geography and public health. Widespread exposure of environment noise is one of the leading risk of human health [1]. In Europe, about 106.1 million disabled-adjusted life years (DALYs) are lost each year due to noise pollution [1]. In China, 35.3% of the complaints received by environmental protection authorities in 2018 are about environmental noise, ranking second after air pollution [2]. In addition to the direct damage to the auditory organs, the health impacts of noise also include non-auditory health hazards such as sleep disturbance, cardiovascular disease, hypertension and mental disorders [3–6]. Noise annoyance is considered to be the most widespread and recognized health impact of environmental noise [7]. Numerous studies have shown that noise annoyance is not only a psychological side effect but also an important mediating factor that induces hypertensive cardiovascular and cerebrovascular diseases and ultimately leads to adverse physiological health effects [8, 9]. Therefore, disentangling the relationship between environmental noise and annoyance can help inform the formulation of health-promoting urban planning and environmental management policies.

Environmental noise annoyance refers to an individual’s negative emotional and cognitive response to the repeated disturbance of intended activities due to noise source over a certain time period [10]. According to the primary cause-effect sequence of noise and emotional/cognitive response, three levels of annoyance were proposed by Porter et al. [11] based on different time frames: immediate annoyance, short-term annoyance and long-term annoyance. Immediate annoyance refers to the direct or immediate disturbances by noise, such as awakening, breaking up a conversation or reading, and other physiological responses. Short-term annoyance pertains to the total effects of a short time span (such as several hours or one day after), while long-term annoyance is the general feeling of noise that is formed from an accumulation of acute or short-term responses to noise. The relationships between immediate annoyance, short-term annoyance and long-term annoyance are interactive. For instance, several studies have shown that there is a correlation between short-term and long-term annoyance [12–15]. In previous studies, noise annoyance is generally considered as a long-term effect of noise based on the retrospective recall of general feelings towards long-term noise exposure [16, 17], which is limited by recall bias and ignored the effects of the geographic context of the range of individual mobility. Geographic ecological momentary assessment (GEMA) is proposed to link momentary experience with the individual’s geographic context [18, 19].

Exposure–response curves are often been used to show the relationship between measured noise levels and noise annoyance [16, 20]. Studies have shown dose-dependent effects of noise on annoyance [21], but some studies have found that measured noise levels explain only 10%–15% of the variations in people’s ratings of annoyance [22], and other factors also need to be considered.

Non-acoustical variables such as person-related factors [23, 24] and activity-related factors [25–28] are found to play important roles in explaining noise annoyance. Note that the acoustic environment as perceived or experienced and/or understood by a person or people, called the soundscape by the International Organization for Standardization (ISO), is constituted in particular physical and social spaces [29]. Therefore, self-reported feelings such as noise annoyance are not only based on the acoustic environment but also affected by the spatio-temporal interrelationships among people, activities and various features of the places in the physical space [30]. Through a systematic review of relevant papers from 2009 to 2019, Torresin et al. [31] summarized the factors affecting people’s acoustic perceptions of the indoor environments of residential buildings from the aspects of acoustic factors, urban context, individual factors, environmental factors and survey situation. Besides the individual attributes of gender, age [32], education level [33], income [24], marital status, housing type [34], physical and mental health [35, 36], and noise sensitivity [37–39], activity-related factors such as activity type, activity location [40] and companions can also significantly affect self-reported noise annoyance. Moreover, different activity, travel, social, and temporal contexts significantly influence people’s perceived noise and psychological stress [28, 41]. In addition, the influences of activity-space context such as green space [9, 33, 42], sea view [32], access to quiet areas, visual pleasure, construction interval [43], temperature [44], environment pollution [17] and smell [45] on annoyance have also been observed.

Numerous studies have shown an exposure–response relationship between noise and annoyance [46, 47]. However, most previous studies evaluated people’s noise exposure statically based on their residential locations using field measurements and model simulations. There are two major issues with this approach. On one hand, annoyance is the result of noise interfering with human activities such as working, resting, sleeping, and conversations [48], which are performed in different geographic contexts. Therefore, both activity factors and geographic context should be considered in noise annoyance research [23, 45]. The static estimation of noise exposure based on people’s residential location ignores the effects of activity and geographic context other than those of the residential location which can lead to biased conclusions [49]. On the other hand, studies on the subjective evaluations of noise annoyance are mostly based on retrospective noise evaluations that report people’s subjective responses to noise after experiencing noise events for a period of time (e.g., those that lasted for a day or a longer time), with few studies on people’s real-time (momentary) responses to noise exposure. However, some scholars have pointed out that noise annoyance is a transient event and the annoyance may disappear after the transient noise events. But prolonged noise exposure may have some cumulative effects, such as significant physiological changes and health effects [21, 50]. Therefore, it is necessary to study people’s momentary emotional responses to their short-term noise environments and further explore whether their daily acoustic environments influence their momentary responses to noise.

This study aims to explore people’s momentary noise annoyance in various environments associated with different activities and geographic contexts. Specifically, it seeks to answer the following aspects. (1) What is the effect of environmental noise on people’s momentary noise annoyance in different activity contexts? (2) Do the environments of different activity contexts influence people’s momentary noise annoyance? (3) Do people’s daily acoustic environments influence their momentary noise annoyance?

## Methods

### Study area and participants

**Fig. 1:**
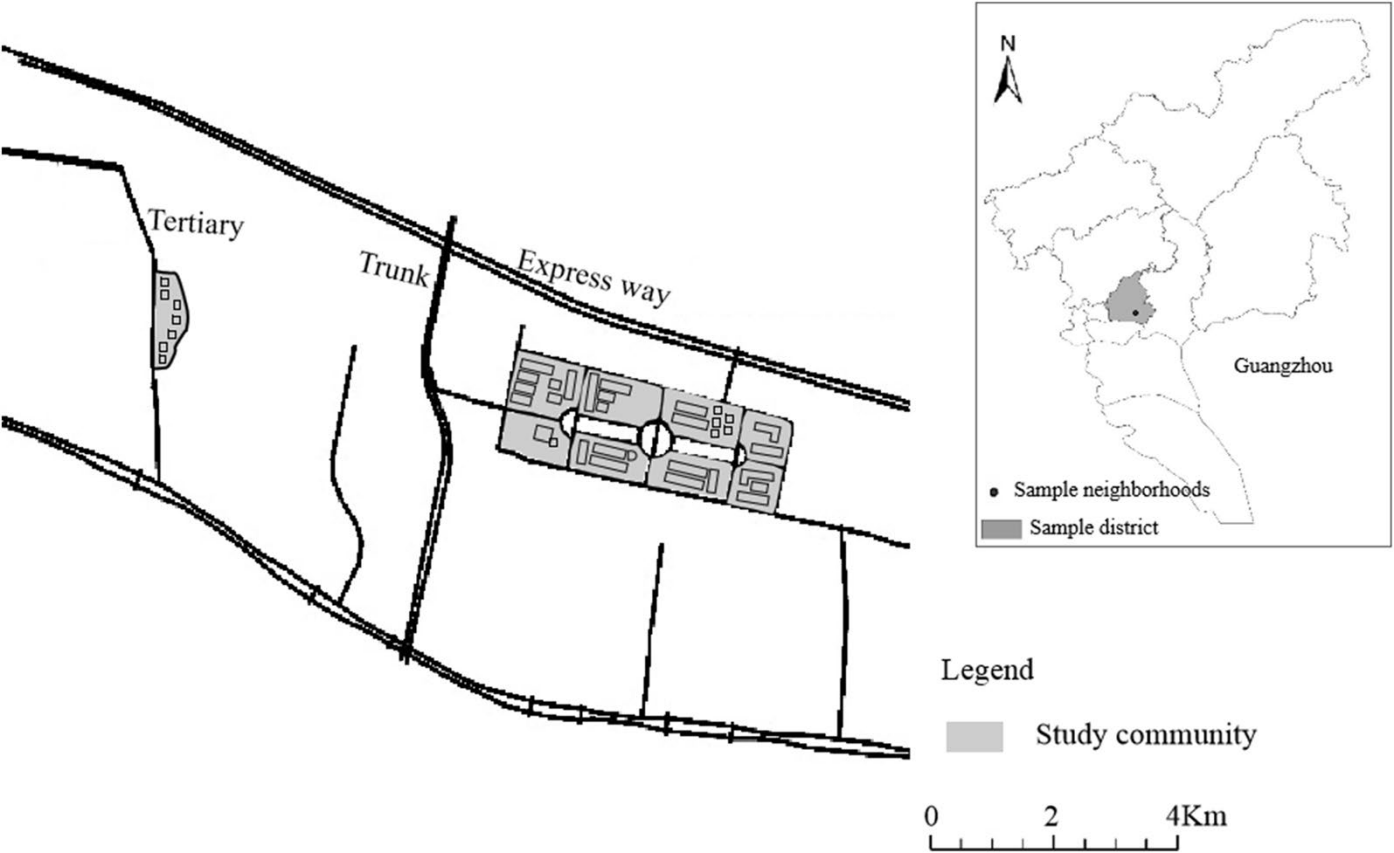
The study area and community

To examine people’s momentary noise annoyance in various activity and geographic contexts, this study used the data collected in a survey of people’s daily activity and environmental exposure in Guangzhou, China from November 2018 to January 2019. This study focus on the Tangxia Street, which is located in the central area of Guangzhou (Fig. 1). Tangxia Street is a large-scale comprehensive residential area covering various housing types, including commercial housing, affordable housing, public rental housing and rental housing of “urban villages”. Using Tangxia Street as a case, we can examine variations in individual noise exposure in different living environments and the relationship between noise exposure, personal noise perceptions and momentary emotions.

The participants of the survey were recruited through random interceptions and introductions by neighborhood committees. Only adults (older than 18) living in the study area were recruited, and each participate were paid a gift worth 200 Yuan (about $30.5) or 250 Yuan (about $38.1) subsidy after completing the survey. This study obtained the consent and support of the community neighborhood committee who informed the residents of the survey by telephone, and introduced the interested residents to us. In addition, posters about the contents of the survey were displayed at the main entrance and exit of the community. The trained investigator conducted random interceptions and introduced more detail of the study for the residents. Prior to the start of the survey, each volunteer was informed in detail of the survey procedures, instruments and data to be collected and signed an informed consent. Finally, a total of 156 participates responded to this study (participates >> participants).

### Survey procedures

The survey has three main parts: the daily activity and environmental health questionnaire survey, the personal environmental exposure assessment, and geographic ecological momentary assessment (GEMA) of environmental perceptions and emotions.

Firstly, the participants completed the daily activity and environmental health questionnaire survey, which collected personal socioeconomic information, self-reported health and environmental noise assessment information. Secondly, they were also trained to using the data collection devices, including the portable noise sensors (SLM-25 Sound Level Meters), GPS-equipped mobile phones, mobile signal detection devices, and portable air sensors (Air Beam, which can record real-time PM_2.5_, temperature and humidity). All the participants were asked to carry the data collection devices for a continuous 48-h period (from 3 a.m. on Sunday to 3 a.m. on Tuesday) to collect the real-time data.

SLM-25 Sound Level Meters (Gain Express Holdings Ltd., HK, China) were used to record participants’ real-time individual noise data, which logged the data at one-minute intervals with the measurement range of 30–130dBA. The SLM-25 instruments meet the standard of IEC61672 Type 2 and ANSI S1.4 Type 2 Sound Level Meter with an accuracy of < 1.5 dBA error. The CEM SC-05 Sound Level Calibrator (Shenzhen Everbest Machinery Industry Co. Ltd., Shenzhen, China) was used to calibrate the SLM-25 instruments at the beginning and end of the survey [41]. Besides, the GPS-equipped smartphones and AirBeam were used to collect PM_2.5_ (Shinyei PPD60PV), temperature and humidity (MaxDetect RH03) [51]. Via Bluetooth, the AirBeam communicated the measurements approximately once a second to the AirCasting Android app, which maps and graphs the data in real time on smartphone. Meanwhile, the smartphone also recorded participants’ GPS trajectories at a frequency of 1 Hz. All the AirBeam equipment were calibrated with the national fixed air monitoring stations (The fitting effect (R^2^) range from 56 to 89%), which was introduced in detail in Zhou's research [52].

**Fig. 2:**
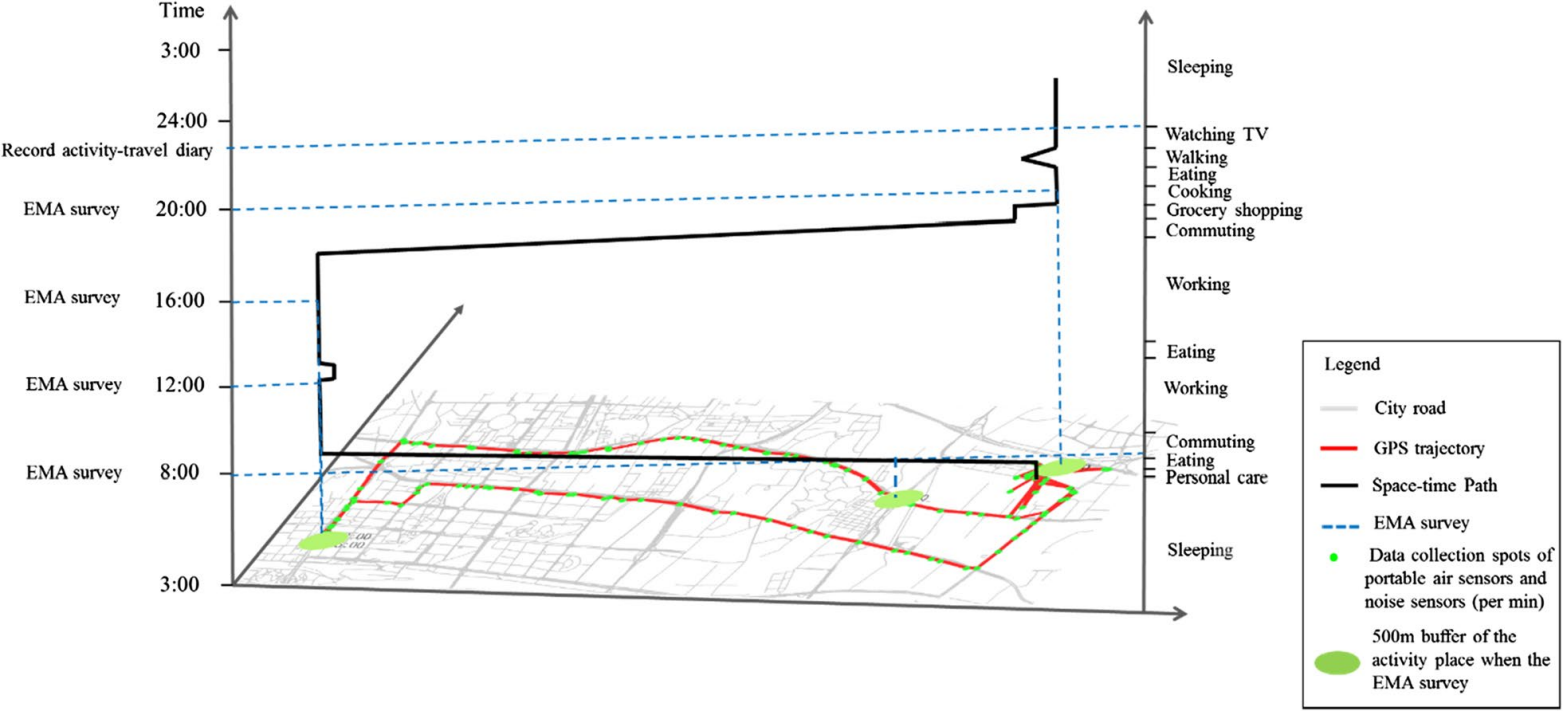
The survey procedures illustrated by the example of one participant’s workday

Then, each participant was requested to respond to the electronic GEMA questions about his/her perception of current noise and annoyance, and the data were sent via the mobile phone at 8:00, 12:00, 16:00 and 20:00 every day. Meanwhile, each participant also carried a mobile signal detection device that recorded the number of mobile phones in the immediate surrounding. This device can sense and record the number of mobile phones within the range of 100 m in real-time. Due to the popularity and portability of mobile devices in cities, the number of mobile phones can objectively reflect the crowdedness within a certain range of the investigated area at a certain time. Last, the participants filled out their activity-travel diaries each night before sleep. Details of each activity and trip including the start and end time, type, location, and companions were recorded through retrospection. Figure 2 illustrates the survey process. During the whole survey process, the status of the mobile devices was remotely monitored to ensure that they were properly functioning and recording the needed data.

After the data collection, the data of the activity-travel diaries were validated by comparing them with the GPS trajectories. Similar to the study by Kou et al. [28], the data of the questionnaire, real-time noise levels and air pollution (PM_2.5_, temperature and humidity), activity-travel diary, GPS track points, and EMA records were integrated based on participants’ unique identifiers and the time when each EMA was submitted. Finally, this article was based on 1046 records of the integrated GEMA data from 138 participants, the other 18 participates were excluded for partial data missing.

### Measures and data analysis

In the EMA survey, the immediate annoyance effects of noise were assessed using the following questions: “To what extent are you currently annoyed by the ambient noise?” Five response categories on a 5-point scale were used: “not at all,” “slightly,” “moderately,” “considerably,” and “very much,” with values of 1–5 respectively. Besides, the original five response categories were recoded into two: “not at all” and “slightly” were recoded to 0 (no or little effect on momentary annoyance), and “moderately,” “considerably,” and “very much” were recoded to 1 (having an effect on momentary annoyance).

Participants’ momentary measured noise was derived with the following steps. First, participants’ activities during each EMA response were recorded in their activity-travel diaries. Noise annoyance is the participant’s response to the repeated disturbance of their intended activities due to noise sources over a certain time period [10]. A-weighted equivalent sound pressure level is a general method adopted by International Organization for Standardization (ISO) to measure noise exposure of individual [53]. It refers to the average value of A sound level according to energy for a certain period of time. Then, A-weighted equivalent sound pressure level (${L}_{Aeq,T}$) between the start of the activity and the GEMA survey was calculated according to the following formula (1).

1 $$L_{Aeq,T} = 10\lg \left( {\frac{1}{T}\mathop \sum \limits_{n = 1}^{T} 10^{{0.1L_{{eq, T_{n} }} { }}} } \right){\text{ dB(A)}}$$

where T represents cumulative time T minutes, ${L}_{Aeq,T}$ is the A-weighted equivalent sound level in total T minutes, ${L}_{eq, {T}_{n}}$ is the A-weighted equivalent sound level at the n minute, which is the reading of the every-minute sound level collected by the portable noise sensors.

Three groups of independent variables were used in the study: (1) the geographic environments of activity places (PM_2.5_, temperature, humidity, crowdedness, green spaces, building density); (2) daily acoustic environments (participants’ evaluations of noise and the objectively measured noise); and (3) individual and activity attributes (e.g., gender, age, income, education, marital status, employment status, activity type, activity duration, activity location). How the independent variables were derived is described as follows.

The data of the geographic environments of activity places were either obtained directly by the sensors or measured using buffer areas. Real-time levels of PM_2.5_, temperature and humidity were recorded by the air pollutant sensors. The momentary PM_2.5_, temperature and humidity were calculated from the average values between the start of the activity and the GEMA survey. Crowdedness in the immediate surrounding was measured by the average number of detected mobile phones within ten minutes of responding the EMA survey, by using the mobile signal detection devices. The environmental features include green spaces and building density were assessed by the amounts or values of each of the environmental features inside a buffer area of 500 m (6–8 min’ walking distance) around for participants’ current location (Fig. 2). The amount of green spaces was assessed using LANDSAT7 satellite images to calculate the Normalized Difference Vegetation Index (NDVI) at a 30 m × 30 m spatial resolution. The value of the NDVI is between − 1 and + 1, and a higher value means a higher density of vegetation [54]. Negative NDVI values were removed as they mean the ground is covered by cloud or water. Building density was represented by the ratio of the building surface area to the area of the 500 m buffer zone.

Daily acoustic environments were measured by participants’ evaluations of noise and objectively measured noise. Participants’ evaluations of the daily acoustic environments of their residential neighborhoods were assessed by their answers to the question: “Has your community had significant noise pollution in the last six months?” The answers to this question were “none at all,” “small,” “medium,” “obvious,” and “very serious,” with values of 1 to 5 respectively. Besides, the equivalent sound level of the two survey days (${\mathrm{L}}_{Aeq,48h}$) of each participant was calculated as the objectively measured daily acoustic environment. We assumed that participants’ activity-travel patterns and urban noise distribution are relatively stable in time and space, and the noise exposure levels of participants during the survey correlate with and thus can represent their daily noise exposure level to a certain extent.

The variables of individual and activity attributes were collected separately by the environmental health questionnaire and activity-travel diaries. The individual attributes include gender, age, education level, marital status, employment status, monthly income, physical health and mental health. Participants’ mental health was evaluated by the World Health Organization's Five Well-Being Indexes (WHO-5) [55], which has a total score of 0 to 25. Specifically, a score of less than 13 indicated that the person’s mental health status is poor. Participants’ physical health was assessed using items 1, 4, and 7 of the MOS 36-Item Short-Form Health Survey [56], which has a total score of 0 to 15. Participants’ activity attributes consist of activity type, activity duration, activity location type, presence of companions during the activity, and the timing of activity. The recorded activities were divided into six categories: sleeping, working, personal and family affairs (such as eating and cleaning), shopping, recreational and social activities, and travel. The location of activities was divided into three categories: home, workplace and others. The timing of activity represented the EMA assessment at “8:00”, “12:00”, “16:00”, “20:00”.

To answer the three questions raised above, the analysis included several steps. First, a descriptive statistical analysis was performed. Then, three hierarchical logistic models (HLMs) were estimated to examine the relationships between noise level and individual noise annoyance. HLMs are commonly used in nested data analysis where the dependent variable is categorical. In this study, each participant repeatedly responded to the EMA surveys at multiple time points. Thus, the data based on the EMA, activity attributes, daily acoustic environments and the environmental attributes of the activity places were nested within individuals. HLMs can reveal the differences in environment noise exposure–response among different activity contexts between individuals (Level 1) and within individuals (Level 2), as shown in formulas (2)–(4).

Level 1 model (activity context level):

2 $${\text{Logit}}\left[ {{\text{P}}\left( {M_{ij} = 1} \right)} \right] = \beta_{0j} + \beta_{kj} X_{kij} + \varepsilon_{ij}$$

Level 2 model (individual-level):

3 $$\beta_{0j} = \alpha_{0j} + \beta_{uj} X_{uj} + \pi_{j}$$

The total model:

4 $${\text{Logit}}\left[ {{\text{P}}\left( {M_{ij} = 1} \right)} \right] = \alpha_{0j} + \beta_{kj} X_{kij} + \beta_{uj} X_{uj} + \varepsilon_{ij} + \pi_{j}$$

where the dependent variable $M_{ij}$ represents the noise annoyance of participant j (j = 1, …, 138) when responding to the ith EMA survey (i = 1, …, 8). $M_{ij}$ is a dichotomous variable; $M_{ij}$ = 1 represents having an effect on momentary annoyance, and $M_{ij}$ = 0 represents no influence on momentary annoyance. ${\text{P}}\left( {M_{ij} = 1} \right)$ represents the probability of having an effect; $\varepsilon_{ij}$ and $\pi_{j}$ are the random effects of the activity and environment level and the individual level respectively, and they are normally distributed. $\beta_{0j}$ is the random intercept of activity and environment level; $\beta_{kj}$ (k = 1, …, 12, the number of activity and environmental attributes) is the impact of activity- and environment-level variables on noise annoyance; $\alpha_{0j}$ is the random intercept of individual level; $\beta_{uj}$ (u = 1, …, 8, the number of individual attributes) is the impact of individual-level variables on noise annoyance. $X_{kij}$ and $X_{uj}$ are the variables of at the activity context level and the individual level.

Three HLM models were estimated with noise annoyance as the dependent variable. The null model was used to determine whether there were significant intra-individual differences in noise annoyance response. The intraclass correlation coefficient (ICC) was estimated by formula (5) as the ratio of the between-individual variance and the total variance [57–59]. Model 1 was fitted with the activity context level variables and considers the effect of the geographical environment of activity space. Then the variables of daily acoustic environments were added to Model 2. The variables of activity attributes were included in both Model 1 and Model 2 as control variables. All the models were estimated in HLM version 6.08 using maximum likelihood estimation, and a two-tailed T-test was used to assess the regression coefficients.

5 $${\text{ICC}} = \frac{{\sigma _{{between}}^{2} }}{{\sigma _{{between}}^{2} + \left( {{\raise0.7ex\hbox{${\pi ^{2} }$} \!\mathord{\left/ {\vphantom {{\pi ^{2} } 3}}\right.\kern-\nulldelimiterspace} \!\lower0.7ex\hbox{$3$}}} \right)}}$$

where ${\sigma }_{between}^{2}$ is the variance between individuals.

Finally, a nonlinear regression model was constructed by random forest to further examine the nonlinear relationship between environmental noise and real-time annoyance. As many previous studies have shown, although there is a dose–response relationship between noise and annoyance, the disturbance and annoyance to activities could significantly increase when the noise level exceeds a certain threshold [60]. The random forest model was used to further examine such complex variations in the relationships between momentary annoyance and an increase in the noise level.

## Results

### Participants’ characteristics

The individual attributes of the participants were summarized in Table 1. There were slightly more women than men in the sample, half of whom were between the age of 31 and 45, and 41.3% had a monthly personal income below 3000 Yuan (about $438.9). 25.4% of the participants had poor mental health and 10.9% had hypertension. 6.7%, 18.1%, 41.6%, 22.7% and 10.9% of them were sleeping, working, dealing with personal or family affairs, shopping or participating in recreational or social activities, and traveling respectively at the time of the EMA prompts. The average duration of the activities they conducted was 77.2 min. Moreover, most participants responded to the EMA questions at home (58.1%) and work (18.5%). The average equivalent sound level at the EMA surveys was 55 dB (A). However, most participants felt no noise (65.7%) or were not bothered by noise (77.8%).

**Table 1:** Descriptive statistics of all variables

Variable (N = 138)	%/Mean	Variable (N = 1046)	%/Mean
Individual attributes		Activity attributes	
Gender (%)		Activity type (%)	
Male	47.10	Sleeping	6.70
Female	52.90	Work	18.10
Age (years) (%)		Personal or family affairs	41.60
19–30	19.60	Shopping, recreation and social activities	22.70
31–45	50.70	Travel	10.90
> 46	29.70	Activity duration (min)	77.2
Education level (%)		Companions (%)	
Senior high school or lower	42.00	Yes	64.20
Technical secondary school/ bachelor degree	45.70	No	35.80
Master degree or higher	12.30	Activity location type (%)	
Hukou (%)		Home	58.10
Guangzhou	74.60	Workplace	18.50
Non-Guangzhou	25.40	Others	23.40
Marital status (%)		The timing of activity (%)	
Married	76.10	8:00	24.80
Other	23.90	12:00	25.30
Employment status (%)		16:00	24.80
Full-time employment	59.40	20:00	25.10
Others	40.60		
Personal monthly income (Yuan) (%)		Immediate annoyance and noise	
0–3000	41.30	Nosie annoyance (%)	
3001–6000	32.60	Having effect	22.20
> 6,000	26.10	Having no effect	77.80
Mental health score (%)		Noise level	
< 13	25.40	${\mathrm{L}}_{Aeq,48h}$ within two days (dB (A))	51
13–25	74.60	${\mathrm{L}}_{Aeq,T}$ during the activity (dB (A))	55
Physical health score	10.8		

### The relationships between environmental noise and momentary annoyance

**Fig. 3:**
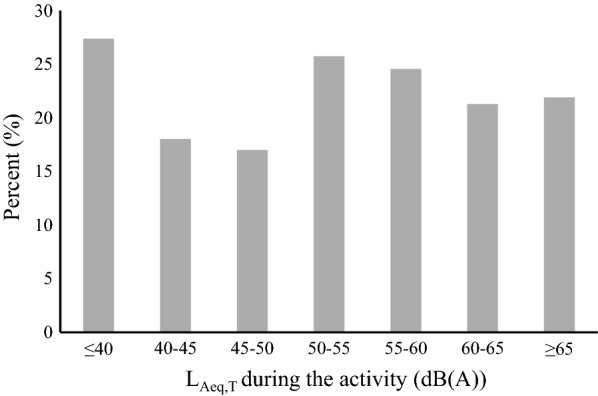
The percentage of participants who felt annoyed at different noise levels

Figure 3 shows the percentages of participants who felt annoyed at different noise levels. Note that these percentages were not linearly related to the equivalent sound level. When the noise level was less than 40 dB (A), the percentage of participants who felt annoyed was at the maximum 27.4%, while when the noise value was 40–50 dB (A), the percentage of participants who felt annoyed was at the minimum 18.0%. Thus, the relationship between noise and annoyance may also be affected by other factors and needs further analysis.

### The influence of geographic context on participants’ noise annoyance

We further analyzed the relationship between noise level and annoyance using a hierarchical binary logistic model after controlling individual and activity variables. The intraclass correlation coefficient (ICC) was 0.292 (ICC ≥ 0.138), which meant that individual attributes explain 29.2% of the variation in noise annoyance. Thus, it is necessary to consider the individual level. But as shown in Table 2, the influence of the geographic environments of activity places on participants’ noise annoyance was insignificant. After considering the geographic environments of activity places, there was a positive but insignificant relationship between noise level and the number of people who felt annoyed by noise (Model 1, OR = 1.14, P > 0.1).

**Table 2:** The hierarchical binary logistic regression models of noise and annoyance

Variables	Model 1	Model 2	Variables	Model 1	Model 2
	OR/(90% CI)	OR/(90% CI)		OR/(90% CI)	OR/(90% CI)
Individual attributes			Activity location (Reference: home)		
Gender (female = 0)	0.87 (0.51, 1.5)	0.89 (0.54, 1.48)	Workplace	1.28 (0.62, 2.67)	1.36 (0.64,2.91)
Age	0.99 (0.74, 1.32)	1.01 (0.77, 1.32)	Others	0.78 (0.43, 1.39)	0.75 (0.41,1.36)
Education level (Reference: Senior high school or lower)			Companions (No = 0)	0.94 (0.66, 1.33)	0.95 (0.67,1.36)
Technical secondary school/bachelor degree	2.20 (1.15, 4.2)**	1.67 (0.88, 3.18)	The timing of activity (Reference: 8:00)		
Master degree or higher	0.67 (0.28, 1.65)	0.56 (0.22, 1.40)	12:00	0.97 (0.67, 1.42)	0.94 (0.64,1.38)
Income (Reference: 0–3000Yuan)			16:00	1.27 (0.91, 1.77)	1.27 (0.90,1.78)
3001–6000	0.90 (0.48, 1.68)	1.05 (0.56, 1.98)	20:00	0.95 (0.61, 1.47)	0.91 (0.58,1.43)
> 6000	2.57 (1.25, 5.26)**	3.27 (1.41, 7.59)***	The geographic environment of activity place		
Marital status (Married = 0)	1.45 (0.77, 2.73)	1.23 (0.69, 2.21)	PM_2.5_ (μg/m^3^)	0.98 (0.81, 1.18)	1.01 (0.83,1.23)
Work (Full-time employment = 0)	1.22 (0.55, 2.72)	1.12 (0.56, 2.26)	Temperature (℃)	1.10 (0.94, 1.29)	1.15* (0.98,1.37)
Mental health (Reference: 13–25)	1.03 (0.55, 1.96)	0.90 (0.52, 1.55)	Humidity (%)	1.02 (0.84, 1.22)	1.04 (0.85,1.27)
Physical health	1.26 (0.93, 1.7)	1.17 (0.86, 1.57)	Green space	0.96 (0.80, 1.15)	0.96 (0.79,1.16)
Activity attributes			Building density	0.97 (0.82, 1.14)	0.96 (0.81,1.14)
Activity type (Reference: Sleeping)			Population density	0.89 (0.75, 1.06)	0.89 (0.74,1.06)
Work	0.62 (0.30, 1.30)	0.57 (0.27, 1.22)	Daily acoustic environment		
Personal or family affairs	0.69 (0.39, 1.22)	0.67 (0.38, 1.20)	L_Aeq_,_48h_		0.74 (0.58,0.93)**
Shopping, recreation and social activities	0.77 (0.40, 1.50)	0.76 (0.39,1.48)	Subjective evaluation of community noise		1.44 (1.12,1.85)***
Travel	1.22 (0.54, 2.73)	1.27 (0.55,2.90)	Sound level (dB (A))	1.14 (0.97, 1.35)	1.20 (1.01,1.44)**
Activity duration	0.94 (0.80,1.10)	0.93 (0.79,1.10)			

The index of PM, temperature, humidity, NDVI, building density, population density and ${\mathrm{L}}_{\mathrm{Aeq},48\mathrm{h}}$ have been standardized. Variance inflation factors (VIF < 5)

*OR* odds ratio, *CI* confidence interval

*P < 0.1, **P < 0.05, ***P < 0.01

### The influence of the daily acoustic environment on participants’ noise annoyance

In Model 2, we considered both the geographic environments and daily acoustic environment. The results showed that there was a significant positive relationship between participants’ evaluation of noise near their residence and their annoyance levels in response to environmental noise (Model 2, OR = 1.44, P < 0.01). However, the relationship between measured environmental noise and annoyance was significant and negative (Model 2, OR = 0.74, P < 0.05). This indicated that participants with higher exposure to environmental noise in their daily lives were less likely to experience annoyance due to noise. However, participants who were more dissatisfied with the acoustic environment of the residential area were more likely to be annoyed.

Besides, participants’ noise annoyance was also related to the individual and activity attributes. For example, participants with a technical secondary school/bachelor degree were more likely to be annoyed than people with lower educational attainment (Model 1, OR = 2.20, P < 0.05). Also, participants with higher income were easier to be annoyed by noise than those with lower income (Model 1, OR = 2.57, P < 0.05; Model 2, OR = 3.27, P < 0.01). In particular, physical health had an impact on environmental noise annoyance: participants who self-reported better physical health were more likely to feel annoyed, but the effect was not statistically significant (The OR of Model 1 and Model 2 are 1.36 and 1.30, P > 0.1). For the activity attributes, only activity type and activity location influence noise annoyance. Compared with sleeping, participants were less likely to be annoyed by noise while working, conducting personal or family affairs, and shopping, recreational and social activities, but the effect was not statistically significant. However, it was easier for participants to feel annoyed when traveling (Model 2, OR = 1.40, P > 0.1). And for activities performed in workplaces compared with at home, the probability of annoyance due to noise was higher (Model 1, OR = 0.78, P > 0.1; Model 2, OR = 0.75, P > 0.1). Besides, the temperature had effects on participants’ annoyance by environmental noise. The higher the temperature was, the more likely participants were annoyed by noise (Model 2, OR = 1.15, P < 0.1).

When both the geographic environments at the activity places and daily acoustic environments were considered, the number of participants who felt annoyed increases significantly with an increase in the noise level (Model 2, OR = 1.20, P < 0.05).

### The nonlinear relationship between environmental noise and annoyance

**Fig. 4:**
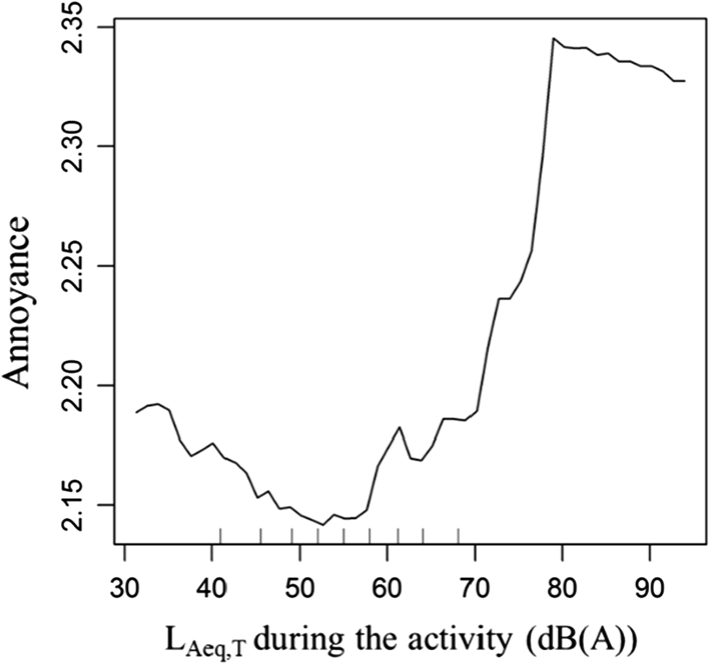
Non-linear effects of noise on real-time annoyance during the EMA survey

To further examine the nonlinear relationship between environmental noise and real-time annoyance, a nonlinear regression model was estimated using the Random Forest method. Based on the above analysis, individual attributes, activity attributes, the geographic environments of activity places and the daily acoustic environments were included as the covariates. Figure 4 shows the partial dependence of real-time annoyance on noise exposure during activity, which indicated the nonlinear and complex relationship between noise and annoyance. Specifically, when the noise level was 45–58 dB (A), the feeling of annoyance was the lowest. When the noise level was from 58 dB (A) to 68 dB (A), the feeling of annoyance increased to a small extent. When the noise level exceeded the value of 70 dB (A), the feeling of annoyance increased greatly with the increase of noise, reaching the maximum value of 78 dB (A) and then basically staying stable beyond this point.

## Discussion

This study is among the first to examine the influence of environmental noise on people’s momentary noise annoyance when they are conducting different activities at different places. The results indicated that the average equivalent sound level of the participants experienced was 55 dB (A), which was associated with the activities they were conducting during the EMA surveys, and only a few participants felt annoyed by noise at this level (22.2%). More importantly, the geographic environments at activity places and the daily acoustic environments of the participants had influence their momentary noise annoyance. Finally, we also observed a nonlinear relationship between environmental noise and participants’ real-time annoyance.

Similar to most studies on noise annoyance, this study found a significant positive relationship between noise and people’s momentary annoyance. After controlling for individual attributes, activity attributes, the geographic environments of activity places and daily acoustic environments, the percentage of participants who felt annoyed significantly increases as the noise level increased. Moreover, only the temperature of activity places had an influence on momentary annoyance that the higher the temperature, the more likely participants were annoyed, which was similar to previous studies [43, 44].

Participants’ daily acoustic environment was also an important factor that affected their momentary noise annoyance. Participants with more serious perceived noise pollution in their residential communities were more likely to feel annoyed by exposure to environmental noise, which is similar to the results of previous research [61]. Participants with higher exposures to measured noise for a long time were less likely to be annoyed. Such a conclusion, on one hand, reflected the importance of individual noise sensitivity to momentary noise annoyance. Noise sensitivity is an individual trait and is generally regarded as an independent variable; that is, highly sensitive participants reported higher levels of annoyance regardless of the measured noise level. A large number of studies have also shown that noise sensitivity can mediate the relationship between noise and annoyance. However, noise sensitivity was not considered in this paper in order to avoid masking the relationship between measured noise and annoyance associated with the perceived noise level. On the other hand, the results also reflected individuals’ self-adaptability to the environment and their preferences for different activity places: people who were exposed to noisy environments for a long time will adapted to the noise environment and tended to report a lower degree of annoyance.

We also observed the nonlinear relationship between environmental noise and momentary annoyance. When the noise level was 45–58 dB (A), the feeling of annoyance was the lowest for different activities. When the noise level exceeded 58 dB (A), the level of annoyance increased slightly at first and then greatly when the noise level exceeded 70 dB (A). The threshold of the effect of noise on momentary annoyance was 58 dB (A) to 78 dB (A). Compared with the five-level annoyance scale of the noise level of Crocker’s [62] research, the noise level of 58 dB (A) was the turning point for the rapid rise of annoyance level, and 68 dB (A) was the critical value for the highest annoyance level. Different from previous studies, when the noise level was in the 32–50 dB (A) range, it was negatively correlated with momentary annoyance, which meant that a lower noise level in this range was associated with a greater level of annoyance. There may be two reasons for this. One is that there are other acoustic factors besides the noise level that also influence annoyance, such as the frequency of the noise. Research has shown that people may complain even when the noise level is within the range mandated by local regulations, and complaints of low-frequency noise from fans, ventilation systems and heat pumps account for 71% of the total [63–65]. A study based on noise exposure to indoor heat pumps/ventilators also found that the incidence of annoyance caused by long-term exposure to low-frequency noise was 15–20%, although the noise level was only 30–50 dB (A) [66]. Nearly 80% of the survey responses in this study were performed indoor, which meant that the kind of noise participants was exposed to may mainly come from electrical equipment in their daily lives. This may also be the reason for the lower average equivalent sound level and the percentage of participants who feel annoyed in this study when compared to other studies.

Based on GPS and real-time sensor technologies and with GEMA surveys, this study collected the spatial and temporal location data of participants and captured the noise environments and geographic contexts of their residences and other activity places, as well as their momentary annoyance. The strengths of this study included the following: (1) The effects of short-term environmental noise on participants’ momentary noise annoyance were analyzed in the context of their current activities; (2) Through the accurate measurement of activity-related environments, the influence of the geographic environment, built environment and social environment of different activity places on noise annoyance was clarified; and (3) The impact of the daily acoustic environment on instantaneous environmental noise emotional was examined and discussed.

Previous studies on the influence of noise based on people’s residences cannot reflect their real-time activity contexts and may face some fundamental methodological problems [49, 67, 68]. The key is the neglect of the influence of spatial and temporal uncertainties on research results, which contributes to the uncertain geographic context problem (UGCoP). On the one hand, individual environmental noise exposure depends on the physical contact between pollutants and individuals, which both vary or move over space and time [69]. We thus need to adopt a dynamic multi-scenario approach to measure individuals’ environmental exposure. On the other hand, people’s responses to environmental context are idiosyncratic [67], and environmental influence is experienced and interpreted by different individuals according to their background and experience. Finally, the effects of environmental noise may have different temporalities. For instance, the health effects of noise would manifest through both real-time annoyance after short-term exposure and cumulative physical and mental health hazards after long-term exposure. The results of this paper also showed that the dose–response relationship between noise and annoyance became obvious after considering the influence of daily acoustic environmental background (Model 2). The results of this study thus also provided further support for the UGCoP.

However, the study also has some limitations. First, the relationship between noise and annoyance is complex and affected by both acoustic and non-acoustic factors. In this study, although individual attributes, activity attributes, the environments of activity sites and daily acoustic environment background factors were considered, some acoustic factors were not considered (e.g., the type of noise source and noise frequency), which may also moderate the effect of noise on annoyance as shown in previous studies [16, 70–73]. Second, noise annoyance is one of the most commonly used indicators for evaluating the health effects of environmental noise exposure. It is generally obtained by questionnaire surveys (based on participants’ subjective evaluations). At present, no objective evaluation method has been established to support the subjective assessment results of questionnaire survey. Therefore, self-report bias may existed in the EMA surveys of this study. Lastly, it should be noted that nearly 80% of the survey responses in this study were made indoor, which may be the reason why a lot of the participants felt annoyed when the noise value was lower (because there is evidence that at the same sound level, indoor noise leads to higher levels of annoyance than outdoor noise) [40, 74].

## Conclusion

Based on real-time individual environment exposure data and geographic ecological momentary assessment (GEMA), this study examined the effects of environmental noise on people’s real-time annoyance in various activity contexts. More specifically, the study analyzed the influence of the geographic contexts of activity places and daily acoustic environment background on participants’ noise annoyance. There are three main conclusions. First, the average equivalent sound level of participants was 55 dB (A) when they responded to the EMA surveys, which met the daytime noise standard of 55 dB (A) in residential area in China’s Environmental Quality Standard for Noise (GB3096-2008) [75]. Second, the geographic environments of active places affected participants’ real-time noise annoyance (e.g., the higher the temperature, the more likely participants are annoyed). Third, the daily acoustic environment also had an effect on individual noise annoyance: participants who were more dissatisfied with the acoustic environment of their residential areas were more likely to be annoyed. However, participants with a higher objective value of daily noise exposure were less likely to be annoyed. Last, after controlling for individual-related and activity-related variables, there was a nonlinear relationship between environmental noise and real-time annoyance, and the threshold of participants’ real-time noise annoyance was 58–78 dB (A).

## Data Availability

The datasets used and/or analysis during the current study are available from the corresponding authors on reasonable request.

## Abbreviations

GEMA: Geographic ecological momentary assessment
EMA: Ecological momentary assessment
WHO: World Health Organization
DALYs: Disabled-adjusted life years
ISO: International Standardization Organization
GPS: Global position system
NDVI: The normalized difference vegetation index
WHO-5: World Health Organization's five Well-Being indexes
HLMs: Hierarchical logistic models
ICC: Intraclass correlation coefficient
OR: Odds ratio
CI: Confidence interval
VIF: Variance inflation factors
UGCoP: Uncertain geographic context problem
